# Investigation of Phase Segregation in *p*-Type Bi_0.5_Sb_1.5_Te_3_ Thermoelectric Alloys by In Situ Melt Spinning to Determine Possible Carrier Filtering Effect

**DOI:** 10.3390/ma14247567

**Published:** 2021-12-09

**Authors:** Dong Ho Kim, TaeWan Kim, Se Woong Lee, Hyun-Sik Kim, Weon Ho Shin, Sang-il Kim

**Affiliations:** 1 Departament of Materials Science and Engineering, University of Seoul, Seoul 02504, Korea; hoo829@uos.ac.kr (D.H.K.); lswprawn245@uos.ac.kr (S.W.L.); 2 Departament of Electrical Engineering and Smart Grid Research Center, Jeonbuk National University, Jeonju 54896, Korea; twkim@jbnu.ac.kr; 3 Departament of Materials Science and Engineering, Hongik University, Seoul 04066, Korea; hyunsik.kim@hongik.ac.kr; 4 Departament of Electronic Materials Engineering, Kwangwoon University, Seoul 01897, Korea; weonho@kw.ac.kr

**Keywords:** thermoelectric, phase segregation, melt spinning, carrier filtering effect

## Abstract

One means of enhancing the performance of thermoelectric materials is to generate secondary nanoprecipitates of metallic or semiconducting properties in a thermoelectric matrix, to form proper band bending and, in turn, to induce a low-energy carrier filtering effect. However, forming nanocomposites is challenging, and proper band bending relationships with secondary phases are largely unknown. Herein, we investigate the in situ phase segregation behavior during melt spinning with various metal elements, including Ti, V, Nb, Mo, W, Ni, Pd, and Cu, in *p*-type Bi_0.5_Sb_1.5_Te_3_ (BST) thermoelectric alloys. The results showed that various metal chalcogenides were formed, which were related to the added metal elements as secondary phases. The electrical conductivity, Seebeck coefficient, and thermal conductivity of the BST composite with various secondary phases were measured and compared with those of pristine BST alloys. Possible band alignments with the secondary phases are introduced, which could be utilized for further investigation of a possible carrier filtering effect when forming nanocomposites.

## 1. Introduction

Thermoelectric technology has attracted attention for its use in solid-state cooling and energy harvesting because it can convert heat directly into electricity. The energy conversion efficiency of thermoelectric materials is limited by the dimensionless figure of merit, *zT* = [S^2^∙*σ/*(*κ_ele_ + κ_latt_*)] × *T*, where *S* is the Seebeck coefficient, *σ* is the electrical conductivity, *κ_ele_* is the electronic thermal conductivity, *κ_latt_* is the lattice thermal conductivity, and *T* is the absolute temperature [1,2,3,4]. Accordingly, a high *zT* value can be achieved by increasing *S*^2^∙*σ* and reducing the thermal conductivities (*κ*_ele_ and *κ*_latt_). However, these thermoelectric parameters are generally interdependent. Therefore, based on a comprehensive analysis of the fundamental mechanisms, thermoelectric materials should be manipulated to achieve optimal thermoelectric properties. In recent years, many approaches have been improved using zT values. Control of the carrier concentration, resonance doping, band engineering, and carrier filtering effects have been suggested for enhancing *S*^2^∙*σ* [5,6,7,8]. However, other strategies exist for reducing thermal conductivity. These include inducing point defects, dislocation arrays, or nanostructures by increasing phonon scattering [9,10,11,12].

Of these approaches, carrier energy filtering can effectively improve *zT* by increasing *S* and *S*^2^∙*σ*. This type of filtering is achieved by energy barriers at heterointerfaces arising from band bending between the thermoelectric matrix and secondary phases [13,14,15], which induce strong energy dependence on the carrier relaxation time. When proper phase segregation is introduced in thermoelectric materials, the carrier energy filtering effect can be achieved, thereby enhancing the thermoelectric performance through low-energy carrier scattering by potential heights formed at heterointerfaces [13,14]. In addition, phonon scattering can be strengthened by the segregated phases to reduce *κ*_latt_.

Experimental evidence of *S* enhancement by the carrier filtering effect has been reported with various thermoelectric nanocomposites. Dou et al. reported an improvement in S of approximately 20%, as compared with that of the Bi_0.5_Sb_1.5_Te_3_ matrix, which originated from the energy filtering of carriers [15]. Even more noticeable enhancements in S were observed in Sb/SbTe nanocomposites by Zhang et al. [16]. Fan et al. showed that the formation of nano-inclusions through melt spinning could lead to favorable conditions for thermoelectric applications [17]. Recently, Jiang et al. reported noticeable maximum *zT* values of 1.56 at 400 K by inducing PbSe nanocomposites with suppressed lattice and bipolar thermal conductivities that effectively inhibit minor charge carriers [18].

In this study, we investigated in situ phase segregation behavior during melt spinning with various metal elements, including Ti, V, Nb, Mo, W, Ni, Pd, and Cu, in *p*-type Bi_0.5_Sb_1.5_Te_3_ (BST) thermoelectric alloys, which could be utilized for further investigation of a possible carrier filtering effect. The possible band alignments with secondary phases are presented with their measured thermoelectric properties.

## 2. Experimental Section

To prepare a set of samples of Bi_0.5_Te_1.5_Se_3_(*M*)_0.1_ (*M* = Ti, V, Nb, Mo, W, Ni, Pd, and Cu), all high-purity elements (Bi (99.999%, 5 N plus), Te (99.999%, 5 N plus), Se (99.999%, 5 N plus), and metal elements) were stoichiometrically synthesized by subsequent conventional melting and quenching techniques. The synthesized samples were blended using a ball-milling process (8000D, SPEX SamplePrep, Metuchen, NJ, USA) for 5 min. We conducted rapid solidification through melt spinning (Cu wheel rotation, 3600 rpm). The molten ingot was sprayed under a pressure of 0.03 MPa in an argon atmosphere. Using an agate mortar, the ribbons from the melt-spinning process were pulverized. Finally, the powders were sintered at 430 ℃ by spark plasma sintering (SPS) for 5 min under a pressure of 50 MPa.

To analyze the crystalline phases of the samples, X-ray diffraction (XRD, D8 Discover, Bruker, Billerica, MA, USA) was performed at room temperature. Then, the temperature-dependent *σ* and *S* parameters were measured simultaneously over the temperature range between room temperature and 480 K using a ZEM-3 measurement system (Advanced-RIKO, Yokohama, Japan) perpendicular to the SPS pressing direction. The *κ* values were also computed from the theoretical density (*ρ_s_*), heat capacity (*C_p_*), and thermal diffusivity (*D*) in the same direction (*κ* = *ρ_s_ × C_p_ × D*). Then, the diffusivities *λ* were measured by the laser flash method (LFA 467, Netzsch, Wittelsbacherstraße, Germany).

## 3. Results and Discussion

### 3.1. Secondary Phase Formation

**Figure 1:**
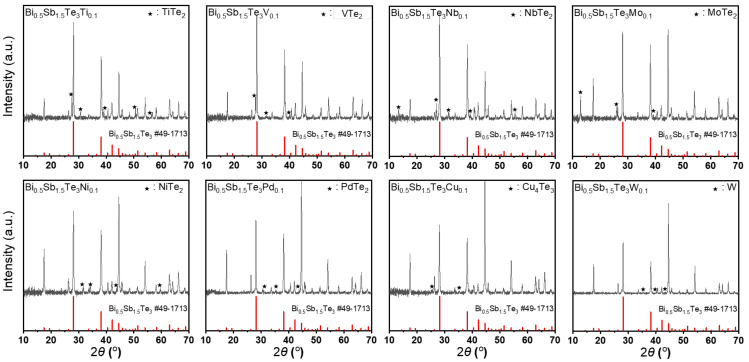
XRD patterns of Bi_0.5_Sb_1.5_Te_3_M_0.1_ (M = Ti, V, Nb, Mo, W, Ni, Pd and Cu).

Figure 1 shows the XRD patterns of the experimental samples of Bi_0.5_Sb_1.5_Te_3_(*M*)_0.1_ (*M* = Ti, V, Nb, Mo, W, Ni, Pd, and Cu). Each diffraction peak commonly showed its own matrix phase (Bi_0.5_Sb_1.5_Te_3_, JCPDs PDF #49-1713) with the secondary phases, which was related to the added metals. The observed secondary phases were TiTe_2_, VTe_2_, NbTe_2_, MoTe_2_, W, NiTe_2_, PdTe_2_, and Cu_4_Te_3_ for various added metal elements (Ti, V, Nb, Mo, W, Ni, Pd, and Cu, respectively). Most secondary phases were formed as dichalcogenides, whereas the addition of Cu caused Cu_4_Te_3_ to form. The addition of W did not lead to the formation of compounds.

### 3.2. Band Bending at Heterointerfaces

**Figure 2:**
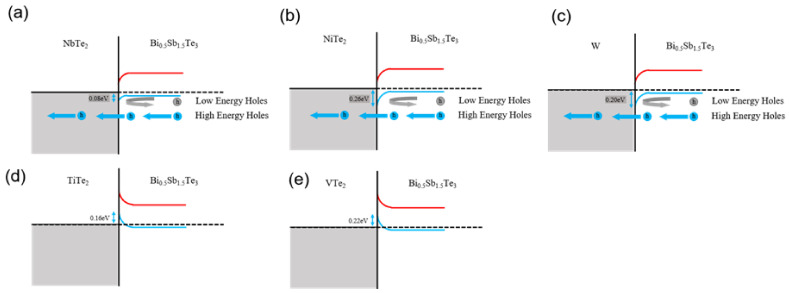
Band alignments at heterointerfaces between Bi_0.5_Sb_1.5_Te_3_ and secondary phases (NbTe_2_, NiTe_2_, W, TiTe_2_, and VTe_2_).

**Figure 3:**
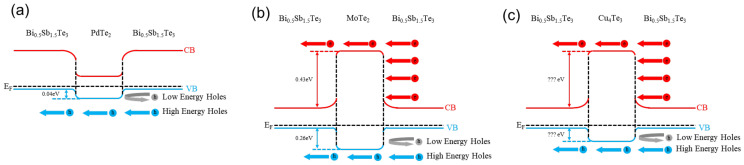
Band alignments in a Bi_0.5_Sb_1.5_Te_3_–*M* heterojunction (*M* = semiconductor).

The band alignment schematics at the heterointerfaces between BST and the secondary phases are shown in Figure 2 and Figure 3. Figure 2 shows the energy bands of the Bi_0.5_Sb_1.5_Te_3_(*M*)_0.1_ (*M* = Nb, Ni, W, Ti, and V) samples, whose secondary phases (NbTe_2_, NiTe_2_, W, TiTe_2_, and VTe_2_) were metallic. The work functions of NbTe_2_, NiTe_2_, W, TiTe_2_, and VTe_2_ were 4.62, 4.44, 4.5, 4.86, and 4.92 eV, respectively [19,20,21]. Possible carrier filtering barriers were formed in Bi_0.5_Sb_1.5_Te_3_Ni_0.1_, Bi_0.5_Sb_1.5_Te_3_Nb_0.1_, and Bi_0.5_Sb_1.5_Te_3_W_0.1_, with NbTe_2_, NiTe_2_, and TiTe_2_ for hole transport, respectively. Their energy barrier heights were 0.08, 0.26, and 0.20 eV for NbTe_2_, NiTe_2_, and TiTe_2_, respectively. For Bi_0.5_Sb_1.5_Te_3_V_0.1_ and Bi_0.5_Sb_1.5_Te_3_Ti_0.1_, no energy barrier was expected with the secondary phases of VTe_2_ and TiTe_2_, respectively.

Figure 3 shows the energy bands of the Bi_0.5_Sb_1.5_Te_3_(*M*)_0.1_ (*M* = Pd, Mo, and Cu) samples, whose secondary phases (PdTe_2_, MoTe_2_, and Cu_4_Te_3_) were semiconducting. The band gap (*E*_g_), Fermi level (*E*_f_), and electron affinity (*χ*) of PdTe_2_, MoTe_2_, and Cu_4_Te_3_ were taken from the literature [19,22,23,24]. The *χ* of Bi_0.5_Sb_1.5_Te_3_ is 4.50 eV and the *E*_g_ is 0.2 eV [25]. Given the band structure of PdTe_2_, the band diagram of Bi_0.5_Sb_1.5_Te_3_Pd_0.1_ is presented in Figure 3a. A possible filtering barrier of 0.04 eV in Bi_0.5_Sb_1.5_Te_3_Pd_0.1_ is shown. In the case of Bi_0.5_Sb_1.5_Te_3_Mo_0.1_, because of the relatively wide *E*_g_ as compared to that of BST, an expected band diagram is given in Figure 3b. It formed a hole barrier of 0.26 eV, whereas the electron filtering barrier reached 0.43 eV. In the case of Bi_0.5_Sb_1.5_Te_3_Cu_0.1_, no band data were available for Cu_4_Te_3_. Because a quantitative illustration of the band diagram was unavailable, the illustration is shown with no quantitative values. Table 1 lists the work functions of *E*_g_ and *χ* for the segregated phases.

### 3.3. Electronic Transport Properties (σ, S, and S^2^∙σ)

**Figure 4:**
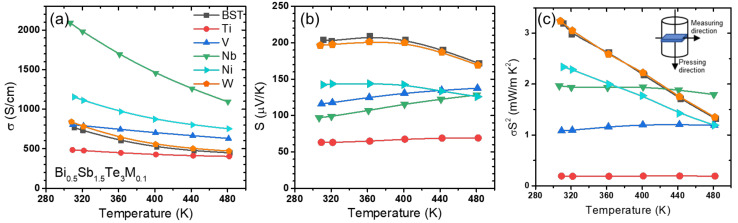
Temperature dependences of the (**a**) electrical conductivity, (**b**) Seebeck coefficient, and (**c**) power factor in Bi_0.5_Sb_1.5_Te_3_*M*_0.1_ (*M* = Ti, V, Nb, Mo and W).

The temperature dependences of *σ* for Bi_0.5_Sb_1.5_Te_3_(*M*)_0.1_ (*M* = Ti, V, Nb, W, and Ni) are shown in Figure 4a. The *σ* value of the pristine BST sample was 767 S/cm at room temperature and decreased to 448 S/cm with increasing temperature. For the Bi_0.5_Sb_1.5_Te_3_Ti_0.1_ and Bi_0.5_Sb_1.5_Te_3_V_0.1_ samples, which did not form energy barriers at the heterointerfaces (Figure 2), the decreasing slope of *σ* with increasing temperature was much lower than that of the pristine BST, whereas the *σ* of Bi_0.5_Sb_1.5_Te_3_Ti_0.1_ and Bi_0.5_Sb_1.5_Te_3_V_0.1_ generally decreased and increased, respectively, as compared with that of the pristine BST. Note that these two samples did not form adequate energy barriers for hole carrier filtering (Figure 2); the work function of TiTe_2_ and VTe_2_ (4.86 and 4.92 eV, respectively) is much larger than the *χ* of the BST matrix (4.50 eV). The *σ* value of Bi_0.5_Sb_1.5_Te_3_Ti_0.1_ showed a lower *σ* value of 484 S/cm at room temperature. For the other samples (*M* = Nb, Ni, and W), the *σ* values all increased, as compared with that of the pristine sample. The *σ* values of Bi_0.5_Sb_1.5_Te_3_Nb_0.1_ and Bi_0.5_Sb_1.5_Te_3_Ni_0.1_ reached their maxima at 2143 and 1154 S/cm at room temperature, respectively. The *σ* value of Bi_0.5_Sb_1._5Te3W_0.1_ was similar to that of the pristine BST at the measured temperatures.

*S* for the Bi_0.5_Sb_1.5_Te_3_(*M*)_0.1_ (*M* = Ti, V, Nb, W, and Ni) samples is given as a temperature-dependent function in Figure 4b. The *S* values of all the samples were suppressed as compared with that of the pristine BST. The BST sample had a peak *S* magnitude of 209 μV/K at 360 K and decreased to 171 μV/K with increasing temperature (at 480 K). At room temperature, *S* decreased to 194, 142, 117, 99, and 63 μV/K for the W-, Ni-, V-, Nb- and Ti- added samples, respectively.

Figure 4c shows the temperature dependence of *S*^2^∙*σ* (power factor) for the Bi_0.5_Sb_1.5_Te_3_(*M*)_0.1_ (*M* = Ti, V, Nb, W, and Ni) samples. Bi_0.5_Sb_1.5_Te_3_W_0.1_ showed very similar power factor values to the pristine BST sample over the entire temperature range. The addition of W did not form the telluride, which generally only affects the electric transport of the BST matrix. Otherwise, the power factors decreased to 2.34, 1.97, 1.08, and 0.19 mW/mK^2^ for the Ni-, Nb-, V-, and Ti-added samples. With the addition of Ti and V, which did not form energy barriers at the heterointerfaces with metallic TiTe_2_ and VTe_2_, *σ* and *S* decreased simultaneously, and the power factor was then reduced considerably. With the addition of Ni and Nb, which did form proper energy barriers at the heterointerfaces with metallic NiTe_2_ and NbTe_2_, *σ* increased significantly, whereas S decreased. As a result, the power factors were moderately reduced. For the Nb-added samples, the power factors at high temperatures of 440 and 480 K were higher than that of the pristine BST. For Ni- and Nb-added samples, further experiments with smaller additions of metal (Bi_0.5_Sb_1.5_Te_3_(*M*)_x_ (*M* = Ni and Nb, *x* ≤ 0.01) were conducted to investigate the possible carrier filtering effect [26]. With a small addition of *x* = 0.01, power factor enhancements were observed with an increase in the effective mass, suggesting that a possible carrier filtering effect occurred.

**Figure 5:**
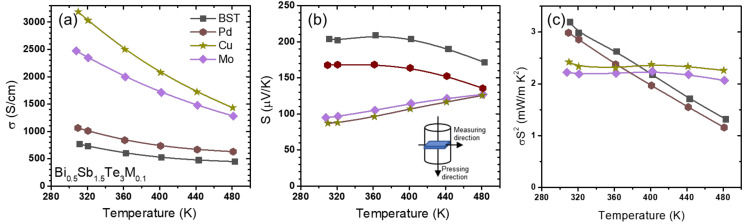
Temperature dependences of the (**a**) electrical conductivity, (**b**) Seebeck coefficient, and (**c**) power factor in Bi_0.5_Sb_1.5_Te_3_*M*_0.1_ (*M* = Ni, Pd and Cu).

The temperature dependences of *σ* for the Bi_0.5_Sb_1.5_Te_3_(*M*)_0.1_ (*M* = Mo, Pd, and Cu) samples, which exhibited semiconducting secondary phases, are shown in Figure 5a. Firstly, significant enhancements in the *σ* values were observed in the Bi_0.5_Sb_1.5_Te_3_(*M*)_0.1_ (*M* = Mo and Cu) samples.

The *σ* value of the Bi_0.5_Sb_1.5_Te_3_Cu_0.1_ sample showed a maximum of 3188 S/cm at 300 K, whereas that of the Bi_0.5_Sb_1.5_Te_3_Mo_0.1_ sample showed a maximum of 2494 S/cm. In the case of the Bi_0.5_Sb_1.5_Te_3_Pd_0.1_ sample, the maximum *σ* value was 1063 S/cm.

For Bi_0.5_Sb_1.5_Te_3_(*M*)_0.1_ (*M* = Mo, Pd, and Cu), S is given as a temperature-dependent function in Figure 5b. The *S* values for Bi_0.5_Sb_1.5_Te_3_ (*M*)_0.1_ (*M* = Mo, Pd, and Cu) were suppressed to 169, 98, and 85 μV/K as compared with 206 μV/K for the pristine BST sample at room temperature.

The temperature dependences of the power factors for the Bi_0.5_Sb_1.5_Te_3_(*M*)_0.1_ (*M* = Mo, Pd, and Cu) samples are shown in Figure 5c. The power factor of Bi_0.5_Sb_1.5_Te_3_Pd_0.1_ decreased slightly as compared with that of the pristine BST. For the Pd- and Cu-added samples, the power factors decreased further, to 2.42 and 2.23 mW/mK^2^, respectively, at room temperature, and greater values were observed at higher temperatures above 400 K.

### 3.4. Thermal Conductivity (κ_tot_, κ_elec_, κ_latt_)

To further investigate the total thermal conductivity (*κ*_tot_) behavior in Bi_0.5_Sb_1.5_Te_3_(*M*)_0.1_ (*M* = Ti, V, Nb, Mo, W, Ni, Pd, and Cu), we determined the *κ*_tot_ values to be mainly binary parts of thermal conductivity, namely, *κ*_ele_ and *κ*_latt_. They were calculated using the following equation:

*κ*_tot_ = *κ*_ele_ + *κ*_latt_ (1)

The *κ*_ele_ values were calculated using the Wiedemann–Franz equation, as follows:

*κ*_ele_ = *L* × *σ* × *T* (2)

where *L* is the Lorenz number (calculated as L = 1.5 + exp(−$\left|S\right|$/116)). *L* and *S* are treated as units in terms of 10^−8^ WΩK^−2^ and μV/K, respectively [27].

**Figure 6:**
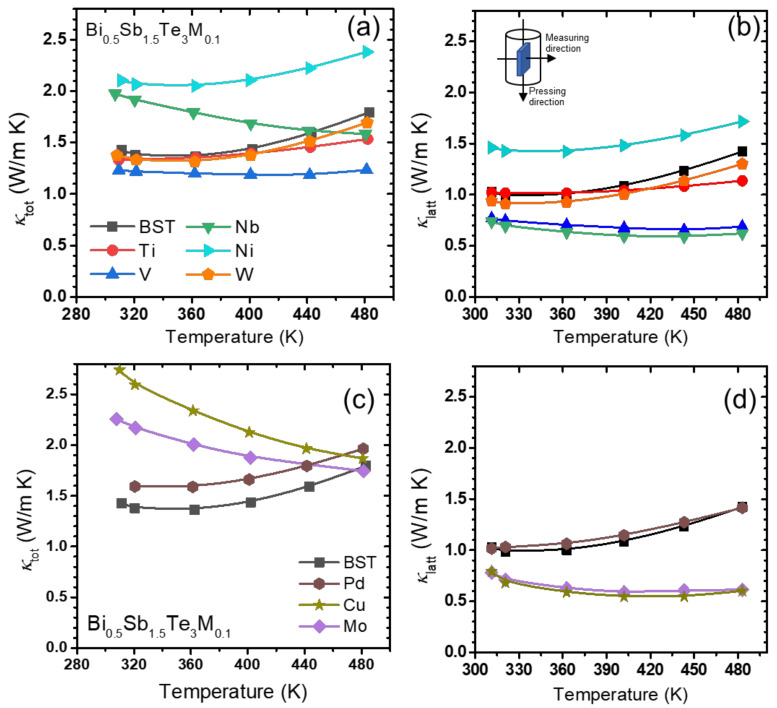
(**a**) *κ*_to*t*_ and (**b**) *κ*_latt_ as a function of temperature for Bi_0.5_Sb_1.5_Te_3_*M*_0.1_ (*M* = Ti, V, Nb, Ni and W); (**c**) *κ*_tot_ and (**d**) *κ*_latt_ as a function of temperature for Bi_0.5_Sb_1.5_Te_3_*M*_0.1_ (*M* = Ni, Pd and Cu).

The *κ*_tot_ and *κ*_latt_ values for the Bi_0.5_Sb_1.5_Te_3_(*M*)_0.1_ (*M* = Ti, V, Nb, Ni, and W) samples, as functions of temperature, are shown in Figure 6a,b, respectively. As shown in Equation (1), we computed the *κ*_latt_ values by subtracting the *κ*_ele_ values (which were calculated in advance) from the *κ*_tot_ values. The *κ*_latt_ values of the standard BST sample were increased from 0.99 to 1.42 W/mK as the measuring temperature increased. For the Bi_0.5_Sb_1.5_Te_3_Ni_0.1_ sample, *κ*_tot_ and *κ*_latt_ increased. *κ*_latt_ was significantly reduced for the V- and Nb-added samples. The addition of W did not form the telluride, which seemed to not affect the thermal conductivity of the BST matrix much.

The *κ*_tot_ and *κ*_latt_ values, as functions of temperature, for the Bi_0.5_Sb_1.5_Te_3_(*M*)_0.1_ (*M* = Mo, Pd, and Cu) samples are shown in Figure 6c,d. For the Bi_0.5_Sb_1.5_Te_3_Pd_0.1_ samples, the general behavior of *κ*_tot_ and *κ*_latt_ with temperature were relatively similar to that of the pristine BST. Bi_0.5_Sb_1.5_Te_3_(*M*)_0.1_ (*M* = Cu and Mo) with higher *σ* values (Figure 5a) exhibited a much higher *κ*_tot_ and showed a gradual decrease with increasing temperature. The *κ*_latt_ for Bi_0.5_Sb_1.5_Te_3_(*M*)_0.1_ (*M* = Cu and Mo) was much lower than that of the pristine BST. In all the samples that formed tellurides, except for the Nb- and Pd-added samples, some degrees of reduction in *κ*_latt_ were shown due to the presence of secondary phases, as observed in Figure 6 [28]. However, adding Nb or Pd, which form NbTe_2_ and PdTe_2_, respectively, increased the *κ*_latt_, or had little effect. At this stage, these different results cannot be elaborated. Further investigation into the possible carrier filtering effects of smaller amounts of Nb- and Pd-added Bi_2_Te_3_-based alloys showed a small degree of reduction in *κ*_latt_ [26,29].

### 3.5. Thermoelectric Figure of Merit zT

**Figure 7:**
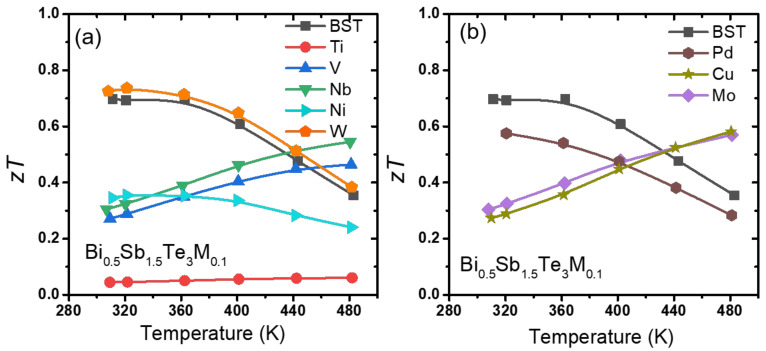
Figure of merit *zT* of (**a**) Bi_0.5_Sb_1.5_Te_3_*M*_0.1_ (*M* = Ti, V, Nb, Mo and W) and (**b**) Bi_0.5_Sb_1.5_Te_3_*M*_0.1_ (*M* = Ni, Pd and Cu).

All the measured values of *S*, *σ*, and *к*_tot_ for all the specimens were used to determine the figure of merit *zT*. The figure of merit zT values are shown in Figure 7a,b. In Figure 7a, the Ti-added samples, which showed a significantly reduced power factor due to the simultaneous reduction of *σ* and *S*, exhibited a considerably reduced *zT*. For the Ni-added sample, a lower *zT* was observed under all temperatures, which was mainly due to the increased *κ*_tot_ and *κ*_latt_. Therefore, the Bi_0.5_Sb_1.5_Te_3_(*M*)_0.1_ (*M* = Ti and Ni) samples showed a lower *zT* over the entire temperature range. For the Bi_0.5_Sb_1.5_Te_3_(*M*)_0.1_ (*M* = V and Nb) samples, the *zT* at low temperatures decreased, whereas that at a high temperature (480 K) exhibited a slightly higher value as compared with the *zT* values of the pristine BST. The addition of W did not form any chalcogenides, which seemed to affect the thermal conductivity of the BST matrix. It showed an improvement in *zT* of approximately 5% as compared with that of the pristine BST. In Figure 7b, the Pd-added samples, which showed a moderately decreased power factor with slightly increased *κ*_tot_ and *κ*_latt_, exhibited a reduced *zT* over the entire temperature range. In the range of 300–440 K, the Bi_0.5_Sb_1.5_Te_3_(*M*)_0.1_ (*M* = Mo, and Cu) samples had lower *zT* values than that of the pristine BST. However, from 440 K to 480 K, the Bi_0.5_Sb_1.5_Te_3_ (*M*)_0.1_ (*M* = Mo, and Cu) samples had slightly higher *zT* values than that of the pristine BST.

## 4. Conclusions

We investigated the in situ phase segregation behavior during melt spinning with various metal elements, including Ti, V, Nb, Mo, W, Ni, Pd, and Cu, in *p*-type Bi_0.5_Sb_1.5_Te_3_ (BST) thermoelectric alloys. The observed secondary phases were TiTe_2_, VTe_2_, NbTe_2_, MoTe_2_, W, NiTe_2_, PdTe_2_, and Cu_4_Te_3_ for various added metal elements (Ti, V, Nb, Mo, W, Ni, Pd, and Cu, respectively). The electrical conductivity, Seebeck coefficient, and thermal conductivity of the BST composite with various secondary phases were measured and compared with those of the pristine BST alloys. The possible band alignments with the secondary phases were introduced, which could be utilized for further investigation of a possible carrier filtering effect when forming nanocomposites.

## Figures and Tables

**Table 1:** Work functions or band gap (*E*_g_) and electron affinity (*χ*) of the precipitates.

Properties	Phases	Work Function or *E*_g_ and *χ* (eV)	Reference
-	Bi_0.5_Sb_1.5_Te_3_	*E*_g_ = 0.2 *χ* = 4.50	
Metallic	TiTe_2_	4.86	[19]
	VTe_2_	4.92	
	NbTe_2_	4.62	
	W	4.5	[20]
	NiTe_2_	4.44	[21,22]
Semi-conducting	MoTe_2_	*E*_g_ = 0.8 *χ* = 4.29	[22]
	PdTe_2_	E_g_ = 0.12 *χ* = 4.36	[23,24]
	Cu_4_Te_3_	unknown	-

## Data Availability

Data available on request.
